# Supplementary material for: Machine learning-based motion tracking reveals an inverse correlation between adhesivity and surface motility of the leptospirosis spirochete
Source: Nat Commun. 2023 Dec 5;14:7703. doi: 10.1038/s41467-023-43366-0 (PMC10697978; doi:10.1038/s41467-023-43366-0)
Supplement: Supplementary file 1 — Supplementary Information [file 41467_2023_43366_MOESM1_ESM.pdf]

Supplementary Information

Title: Machine learning-based motion tracking reveals an inverse correlation between adhesivity and surface motility of the leptospirosis spirochete

Authors: Abe K, Koizumi N, and Nakamura S

Table S1. Bacterial strains used in this study.

Table S2. Summary of experimental data.

Figure S1. Swimming and crawling speeds of the mutant strains on NRK.

Figure S2. Adherence and motility of the mutant strains on MDCK.

Figure S3. Transposon insertion mutants.

Figure S4. Effect of GMM parameters on the detection of paused bacteria.

Figure S5. Dirichlet distribution.

Figure S6. Tracking of GFP-labeled *Leptospira*.

Table S1. Bacterial strains used in this study.

Bacterial strain					Abbr.	Isolated from	Ref.
L. interrogans							
serogroup	Hebdomadis	strain	D-OW16-2K		Hebd-16	Dog	This study
serogroup	Hebdomadis	strain	D-SO21-6K		Hebd-21	Dog	This study
serogroup	Australis	strain	D-FO15-8K		Australis	Dog	This study
serogroup	Icterohaemorrhagiae	strain	RN-TK-AS1707-6		Ictero	Rat	This study
serogroup	Canicola	strain	D-NA10-E1-0K		Canicola	Dog	This study
serogroup	Autumnalis	strain	D-SA11-5K		Autum	Dog	[1]
serovar	Manilae	strain	UP-MMC-NIID		Manilae	Human	[2]
serovar	Manilae	strain	UP-MMC-NIID	*ligA::Tn*	*ΔligA*	-	This study
serovar	Manilae	strain	UP-MMC-NIID	*lenA::Tn*	*ΔlenA*	-	This study

Table S2. Sammary of adhesion and motility assays.

Host<br>Kidney cells	Bacteria<br>*Leptospira*	Adhesion to kidney cells<br>Bacteria/mm^2	Swimming speed<br>v_{SW} (μm/s)	Crawling speed<br>v_{CR} (μm/s)	Crawling ability<br>v_{CR} / v_{SW}
		fields	bacteria	bacteria	
NRK (Rat)	Hebd-16	2899 ± 198 (n = 16)	6.3 ± 0.3 (n = 93)	3.7 ± 0.1 (n = 124)	0.6 ± 0.1
	Hebd-21	2587 ± 221 (n = 18)	12.8 ± 0.3 (n = 200)	7.3 ± 0.5 (n = 100)	0.6 ± 0.1
	Australis	1575 ± 157 (n = 14)	5.5 ± 0.2 (n = 131)	5.0 ± 0.2 (n = 120)	0.9 ± 0.1
	Ictero	2647 ± 177 (n = 12)	9.7 ± 0.0 (n = 329)	6.4 ± 0.2 (n = 432)	0.7 ± 0.0
	Canicola	3268 ± 249 (n = 12)	10.1 ± 0.4 (n = 112)	3.8 ± 0.2 (n = 79)	0.4 ± 0.1
	Autum	1108 ± 66 (n = 16)	3.7 ± 0.1 (n = 220)	6.6 ± 0.3 (n = 119)	1.8 ± 0.1
	Manilae	3582 ± 367 (n = 16)	6.9 ± 0.2 (n = 213)	4.7 ± 0.2 (n = 190)	0.7 ± 0.0
	ΔlenA	1546 ± 117 (n = 20)	5.0 ± 0.2 (n = 161)	5.4 ± 0.2 (n = 116)	1.1 ± 0.1
	ΔligA	1533 ± 111 (n = 15)	6.2 ± 0.2 (n = 114)	4.9 ± 0.2 (n = 108)	0.8 ± 0.1
MDCK (Dog)	Hebd-16	680 ± 133 (n = 14)	4.3 ± 0.2 (n = 113)	5.7 ± 0.3 (n = 117)	1.3 ± 0.1
	Hebd-21	1489 ± 211 (n = 15)	11.7 ± 0.3 (n = 192)	10.2 ± 0.3 (n = 170)	0.9 ± 0.0
	Australis	1760 ± 245 (n = 12)	6.2 ± 0.2 (n = 250)	5.0 ± 0.3 (n = 116)	0.8 ± 0.1
	Ictero	1621 ± 145 (n = 15)	10.2 ± 0.3 (n = 224)	9.6 ± 0.2 (n = 256)	0.9 ± 0.0
	Canicola	1753 ± 252 (n = 15)	7.5 ± 0.3 (n = 103)	8.1 ± 0.5 (n = 110)	1.1 ± 0.1
	Autum	528 ± 102 (n = 20)	5.0 ± 0.3 (n = 116)	7.2 ± 0.2 (n = 190)	1.4 ± 0.1
	Manilae	1005 ± 117 (n = 18)	6.3 ± 0.2 (n = 188)	6.8 ± 0.2 (n = 194)	1.1 ± 0.0
	ΔlenA	1032 ± 173 (n = 18)	3.9 ± 0.1 (n = 177)	5.7 ± 0.2 (n = 123)	1.4 ± 0.0
	ΔligA	N.D.	7.5 ± 0.4 (n = 72)	N.D.	N.D.

See Figure S2 for N.D. of $\Delta ligA$.

Supplementary Note 1: Effect of the loss of functional outer membrane protein genes on adhesin and motility

We measured free-swimming speeds of the wild-type (WT) of *L. interrogans* serovar Manilae and the transposon-insertion mutants (Δ*lenA* and Δ*ligA*) and found that the disruption of the genes significantly affected the swimming speed (Figure S1). Therefore, we used the averaged crawling speeds normalized by the averaged swimming speeds to evaluate the crawling ability of *Leptospira* strains. The value represented by $v_{\mathrm{CR}}/v_{\mathrm{SW}}$ in the main text is the ratio of the speed given by the cell rotation when attached to the host cells to the speed when detached. The errors of $v_{\mathrm{CR}}/v_{\mathrm{SW}}$ were calculated from relative errors of crawling speeds and swimming speeds using a general equation of error propagation: the relative error of $v_{\mathrm{CR}}/v_{\mathrm{SW}}$ is determined by quadratic sum of relative errors of v_{CR} and v_{SW}. As shown in Figure 4a, swimming speeds were different among strains. We thus used $v_{\mathrm{CR}}/v_{\mathrm{SW}}$ to compare the crawling ability among strains in Figure S2, and Figures 5 and 6 in the main text.

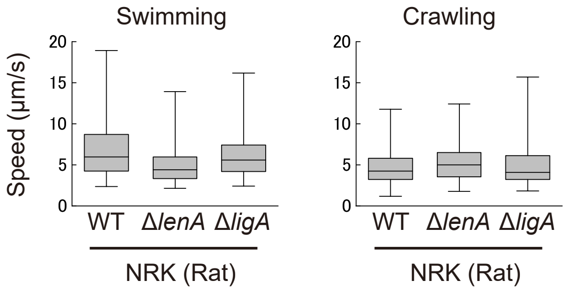

Figure S 1. Swimming and crawling speeds of WT and mutant strains of *L. interrogans* serovar Manilae on NRK. The box-and-whisker plots show the 25th (the bottom line of the box), 50th (middle), and 75th (bottom) percentiles and the minimum and maximum values (whiskers) of the speed data set obtained in each pair. The number of bacteria measured is shown in Table S2.

We also measured adherence and motility of the WT and mutant strains of *L. interrogans* serovar Manilae over the dog kidney cells MDCK. Δ*lenA* retained adhesivity, and its crawling ability was higher than WT (Figure S2). In contrast, very few Δ*lenA* cells attached to MDCK, and thus no motility data were obtained (indicated as N.D. in Figure S2). These results suggest that LenA would be involved in attachment and crawling on MDCK, but LigA likely plays more critical role.

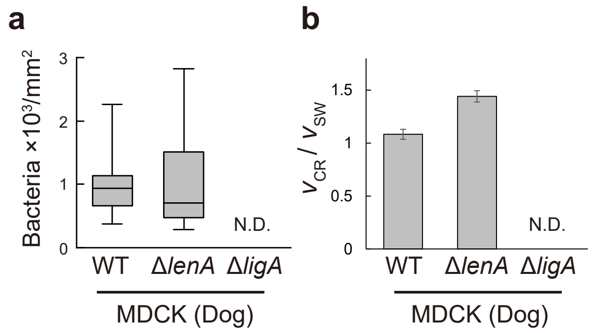

Figure S 2. Adhesion (a) and crawling ability (b) of WT and mutant strains of *L. interrogans* serovar Manilae on MDCK. No data (N.D.) for Δ*ligA* strain. The box-and-whisker plot shows the 25th, 50th, and 75th percentiles (boxes) and the minimum and maximum values (whiskers) of the bacterial-adhesion data set. The number of fields measured is shown in Table S2.

Transposon insertion mutants

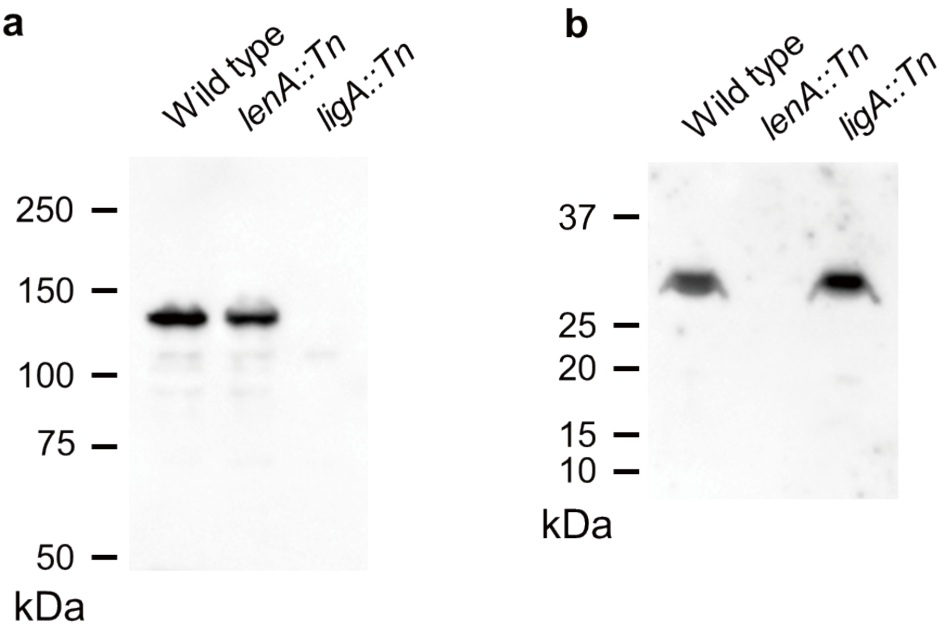

Figure S 3. Immunoblot analysis of whole cell lysates from *L. interrogans* wild-type and transposon insertion mutants (*lenA::Tn* and *ligA::Tn*). *L. interrogans* whole cell lysates (1.5×10^8 cells) were subjected to 5–20% SDS-PAGE and transferred to PVF membrane. The blot was cut into two pieces between 37 kDa and 50 kDa, and the upper and lower blots were probed with anti-LigA (a) and anti-LenA (b), respectively. The reproducibility of the results was confirmed by three independent experiments.

Supplementary Note 2: The theoretical limit of bacteria tracking

As explained in the main text, pixel-value distributions are modeled by the Gaussian mixture model (GMM):

$$\hat{p}(x|H_T, \mathrm{BG}+\mathrm{FG}) = \sum_{\mathrm{m}=1}^{\mathrm{M}} \hat{\pi}_{\mathrm{m}} \mathcal{N}(x; \hat{\mu}_{\mathrm{m}}, \hat{\sigma}^2_{\mathrm{m}}). \tag{1}$$

This model provides M Gaussian distributions, individually weighted by $\hat{\pi}_m$ ($\sum_{m=1}^{M} \hat{\pi}_m = 1$). In the background subtraction, the fractions of foreground (FG) and background (BG) should be determined arbitrarily. If the fraction of FG is defined as c_{fg}, that of BG is $1 - c_{\mathrm{fg}}$. The present study assumes $1 - c_{\mathrm{fg}} \ll c_{\mathrm{fg}}$. Therefore, when $\hat{\pi}_m$ is added from the largest one in descending order until it exceeds $1 - c_{\mathrm{fg}}$, the result indicates the total fraction of BG:

$$B = \arg\min_{\boldsymbol{bg}}\left(\sum_{m=1}^{bg} \hat{\pi}_m > (1 - c_{\mathrm{fg}})\right), \tag{2}$$

where B represents the number of clusters comprising BG distribution. Using B, BG distribution can be modeled by:

$$\hat{p}(x|H_T, \mathrm{BG}) \sim \sum_{\mathrm{m}=1}^{\mathrm{B}} \hat{\pi}_{\mathrm{m}} \mathcal{N}(x; \hat{\mu}_{\mathrm{m}}, \hat{\sigma}^2_{\mathrm{m}}). \tag{3}$$

Consider the case of FG cluster generation when a new pixel value obtained upon object intruding is determined not to belong to any existing clusters. If the object comes to a halt, $\hat{\pi}_m$ is increased with the dwell time according to the following update equation (Eq. 2 in the main text),

$$\hat{\pi}_m \leftarrow \hat{\pi}_m + \alpha(o_m^{(t)} - \hat{\pi}_m) - \alpha c_T, \tag{4}$$

where $c_T = -c_m/T$ (see "Dirichlet distribution" in this Supplementary Information). If $\hat{\pi}_m$ is increased for n frames because of changeless pixel value (i.e., the object stays the same place for n frames), Eq. (4) indicates that $\hat{\pi}_m$ reaches $1 - (1-\alpha)^n$ (assuming $c_T = 0$). When the weight exceeds FG fraction c_{fg}, $1 - (1-\alpha)^n > c_{\mathrm{fg}}$, the FG object is misrecognized. Namely, α and c_{fg} are key parameters determining the theoretical limit n, discriminating moving objects from BG. According to the inequality above, n is determined by:

$$n > \frac{\log(1 - c_{\mathrm{fg}})}{\log(1-\alpha)}. \tag{5}$$

For example, when $c_{\mathrm{fg}} = 0.1$ and $\alpha = 0.001$, n ∼100 frames. This indicates that, even if the object should be FG, it is recognized as BG by halting exceeding 100 frames. Figure S4 shows the effect of n on the FG (bacteria) detection, demonstrating that the increased n enhances noise due to the misrecognition of FG, which prevents motion tracking. Such a problem can be solved by increasing c_{fg} or decreasing α. However, given that α is a modulator decaying the contribution of past data, a decrement of α affects the rate of parameter update adaptation against scene changes. Moreover, the increased c_{fg} could promote the misrecognition of FG as BG. Hence, appropriate values of c_{fg} and α should be verified based on the locomotion property of bacteria. The motion speed is a significant factor to warrant adequate parameter setting. The previous study defined the measured values less than 1 µm/s as non-motile data [3]. Consider a bacterium moving at 1 µm/s. If the cell length is 8 µm (an average length), the bacterium takes 8 s to pass through a pixel. In this study, we recorded bacteria at 0.2 µm/pixel, which is negligibly small against movement distance. Given that the recording frame rate was 30 fps, $n = 240$ is required to detect motion faster than 1 µm/s. Based on these considerations, we set $c_{\mathrm{fg}} = 0.22$ and $\alpha = 0.001$ (n ∼250 frames). Note that bacteria stopping for longer than 8 s will be removed from tracking targets. Hence, our methods can recognize bacteria moving at the speed of >1 µm/s and not stopping for >8 s.

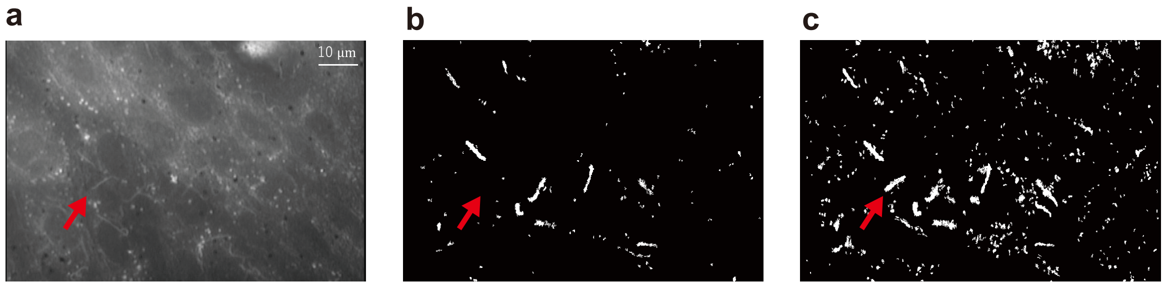

Figure S 4. Effect of GMM parameters on detection of paused bacteria. (a) Raw image. The bacteria indicated by the red arrow has been stopped for more than 100 seconds. The bacterium was not detected by $c_{\mathrm{fg}} = 0.1$ and $\alpha = 0.01$ (n∼10) (b), whereas it was detected by $c_{\mathrm{fg}} = 0.1$ and $\alpha = 0.001$ (n∼105) (c).

Supplementary Note 3: Dirichlet distribution

The Dirichlet distribution is the conjugate prior of the multinominal distribution:

$$P(\vec{x};\vec{\alpha}) = \frac{1}{Z}\prod_{i=1}^{K} x_i^{\alpha_i - 1}, \tag{6}$$

where Z is beta function, $\vec{x} = (x_1, x_2, \sim x_{K-1}, x_K)$ is the stochastic variable ($x_i \geq 0$ and $\sum_{i=1}^{K} x_i = 1$), $\vec{\alpha} = (\alpha_1, \alpha_2, \sim \alpha_{K-1}, \alpha_K)$ is the hyper parameter ($\alpha > 0$). When K events independently occur $\alpha_i - 1$ times for each independently, the distribution gives the occurrence probability of $\vec{x}$.

Consider a three-sided dice numbered from 1 to 3. After rolling the dice many times, the probability at which the dice shows each pip gets close to the same probability: $\vec{x} = (1/3, 1/3, 1/3)$). This means that rolling the dice 30 times results in the occurrence of 10 times for each pip. However, repeating the trial will not show $\vec{x} = (1/3, 1/3, 1/3)$) every time. Namely, though $\vec{x} = (1/3, 1/3, 1/3)$ is the most likely outcome, an infinit number of patterns exist in a range satisfying $\sum_{i=1}^{K} x_i = 1$. This example, assuming that α is a uniform distribution, can be modeled by the Dirichlet distribution with $\alpha > 1$ (Figure S5a). The distribution shows a peak at the center of the triangle, that is $\vec{x} = (1/3, 1/3, 1/3)$. In this study, we assumed that the background (cultured cells) cluster accounts for a major portion of the distribution, and the foreground (bacteria passing through the pixel) is minor. Such strongly biased Dirichlet distribution is given by $0 < \alpha < 1$, biasing x_i to 0 or 1 because of $x_1^{\alpha_1 - 1} x_2^{\alpha_2 - 1} x_3^{\alpha_3 - 1} \to 0$ (Figure S5b). $\alpha_i - 1$ corresponds to $\hat{\pi}_m^{c_m}$ of Eq. (7) in the main text. Therefore, we set $-1 < c_m < 0$.

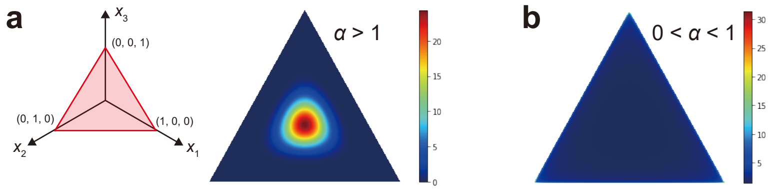

Figure S 5. Dirichlet distributions. (a) The stochastic variables $\vec{x}$ exist within the red triangle because of $\sum_{i=1}^{K} x_i = 1$ (left). The distribution simulated with $\alpha = 10$ (right). (b) $\alpha = 0.9$.

Supplementary Note 4: Tracking of GFP-labeled *Leptospira* by background subtraction method

We used movie data of GFP-labeled *Leptospira* (Figure S6a) to verify whether the motility parameters obtained by the present bacterial tracking based on the background subtraction method are consistent with the results of conventional methods. We also tested the robustness of the background subtraction method against the change in image quality by applying contrast adjustment and noise addition (Gaussian noise with a standard deviation of 10) to the original video (Figure S6b). The image quality was evaluated by the difference between the foreground (bacterium) and background intensities (FG-BG); FG-BG of label-free leptospires measured in this study was roughly 10 - 20 (equivalent to the 7th image in Figure S6b). As a conventional method, center-of-gravity tracking of bacteria binarized at an arbitrary threshold was performed using ImageJ software (National Institutes of Health, MD, USA) (Figure S6c). The tracking of the 7th data was not successful using the conventional method. In contrast, the background subtraction method enabled us to analyze all video data by adjusting the parameters related to the Mahalanobis distance so that pixel data corresponding to bacteria were classified in the foreground distribution (Figure S6d). Here we focused on the crawling speed, the most crucial parameter in this study. At first, the average crawling speed in the trajectory obtained by analyzing the original movie (red box) was determined (v'_{CR}). Next, the average crawling speeds in other trajectories were determined (v_{CR}), and the errors with v'_{CR} were calculated ($|v_{\mathrm{CR}} - v'_{\mathrm{CR}}|$).

Figure S6e shows the results of the analysis of the example data. The conventional method yielded larger velocity errors as the image contrast decreased, while the background subtraction method kept the velocity error to a maximum of about 1 μm/s. We also analyzed 17 traces and their image quality-adjusted data as well as the example data (112 traces in total). The velocity errors were plotted against the image contrast (gray dots in Figure S6f), and the average values of the velocity errors were calculated with a bin width of 20 (red closed circles). In agreement with the result of the example data, for a wide range of image quality, the velocity error is suppressed to around 1 μm/s. Theoretically, our methods can recognize bacteria moving at the speed of >1 μm/s (see the section of The theoretical limit of bacteria tracking). Therefore, we concluded that the difference in the results between the present background subtraction method and the conventional method is not a serious problem.

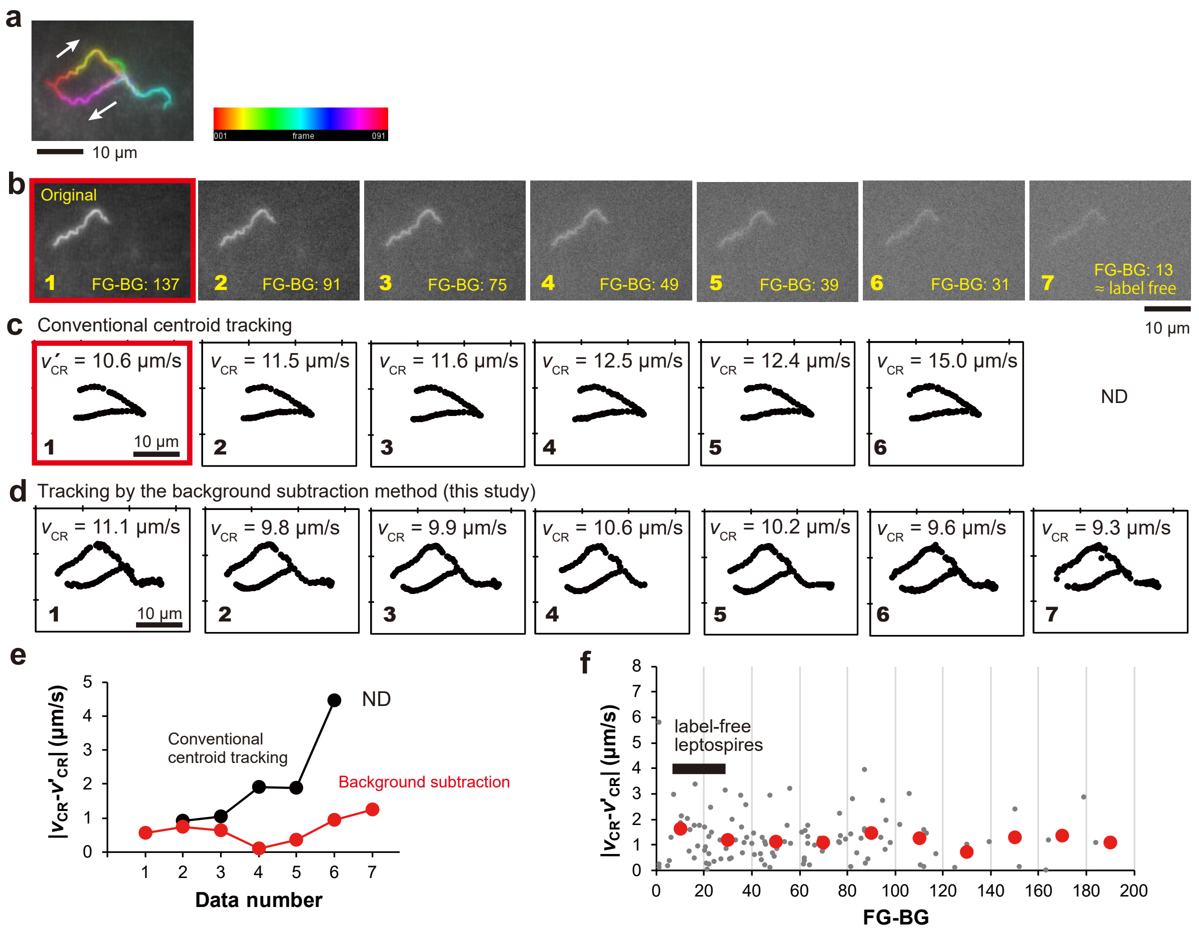

Figure S 6. Tracking of GFP-labeled *Leptospira*. (a) A colored footprint shows a time-lapse record of a crawling leptospiral cell labeled with GFP. The white arrows indicate the direction of crawling. (b) The snapshot of the original video (1) and its contrast-adjusted and noise-added data (2-7). The tracking results obtained by conventional centroid tracking (c) and the background subtraction method developed in this study (d). (e) The velocity errors of the example data. (f) Velocity errors of 17 traces and their image quality-adjusted data.

Supplementary References

[1] Koizumi, N., Muto, M. M., Izumiya, H., Suzuki, M. & Ohnishi, M. Multiple-locus variable-number tandem repeat analysis and clinical characterization of leptospira interrogans canine isolates. *Journal of Medical Microbiology* **64**, 288–294 (2015).

[2] Koizumi, N. & Watanabe, H. Identification of a novel antigen of pathogenic leptospira spp. that reacted with convalescent mice sera. *Journal of medical microbiology* **52**, 585–589 (2003).

[3] Xu, J., Koizumi, N. & Nakamura, S. Crawling motility on the host tissue surfaces is associated with the pathogenicity of the zoonotic spirochete leptospira. *Frontiers in microbiology* **11**, 1886 (2020).
