# Supplementary material for: Machine learning-based motion tracking reveals an inverse correlation between adhesivity and surface motility of the leptospirosis spirochete
Source: Nat Commun. 2023 Dec 5;14:7703. doi: 10.1038/s41467-023-43366-0 (PMC10697978; doi:10.1038/s41467-023-43366-0)
Supplement: Supplementary file 3 — Description of Additional Supplementary Files [file 41467_2023_43366_MOESM3_ESM.pdf]

Description of Additional Supplementary Files:

Supplementary Movie 1: Non-processed video of leptospires over kidney cells (Figure 2a, Step1).

Supplementary Movie 2: Background subtraction (Figure 2a, Step2).

Supplementary Movie 3: Morphological transformation (Figure 2a, Step3).

Supplementary Movie 4: Sorting cells from non-cells (Figure 2a, Step4).

Supplementary Movie 5: The result of tracking crawling bacteria (Figure 2a, Step5).
