# Supplementary material for: Machine learning-based motion tracking reveals an inverse correlation between adhesivity and surface motility of the leptospirosis spirochete
Source: Nat Commun. 2023 Dec 5;14:7703. doi: 10.1038/s41467-023-43366-0 (PMC10697978; doi:10.1038/s41467-023-43366-0)
Supplement: Supplementary file 9 — Reporting summary [file 41467_2023_43366_MOESM9_ESM.pdf]

nature portfolio

Corresponding author(s): Shuichi Nakamura

Last updated by author(s): Oct 31, 2023

Reporting Summary

Nature Portfolio wishes to improve the reproducibility of the work that we publish. This form provides structure for consistency and transparency in reporting. For further information on Nature Portfolio policies, see our Editorial Policies and the Editorial Policy Checklist.

Statistics

For all statistical analyses, confirm that the following items are present in the figure legend, table legend, main text, or Methods section.

n/a	Confirmed	
☐	☒	The exact sample size (*n*) for each experimental group/condition, given as a discrete number and unit of measurement
☐	☒	A statement on whether measurements were taken from distinct samples or whether the same sample was measured repeatedly
☒	☐	The statistical test(s) used AND whether they are one- or two-sided<br>*Only common tests should be described solely by name; describe more complex techniques in the Methods section.*
☒	☐	A description of all covariates tested
☒	☐	A description of any assumptions or corrections, such as tests of normality and adjustment for multiple comparisons
☐	☒	A full description of the statistical parameters including central tendency (e.g. means) or other basic estimates (e.g. regression coefficient) AND variation (e.g. standard deviation) or associated estimates of uncertainty (e.g. confidence intervals)
☒	☐	For null hypothesis testing, the test statistic (e.g. *F*, *t*, *r*) with confidence intervals, effect sizes, degrees of freedom and *P* value noted<br>*Give P values as exact values whenever suitable.*
☒	☐	For Bayesian analysis, information on the choice of priors and Markov chain Monte Carlo settings
☒	☐	For hierarchical and complex designs, identification of the appropriate level for tests and full reporting of outcomes
☒	☐	Estimates of effect sizes (e.g. Cohen's *d*, Pearson's *r*), indicating how they were calculated

Our web collection on statistics for biologists contains articles on many of the points above.

Software and code

Policy information about availability of computer code

Data collection	LightCapture Ver.1.12 (camera software)
Data analysis	Python 3.9.7 (Codes for image analysis and simulation have been deposited in GitHub: https://github.com/shuichi-nakamura-e8/Label-free-bacterial-motion-tracking.git); ImageJ 1.54d; Microsoft Excel for Microsoft 365 MSO (version 2308)

For manuscripts utilizing custom algorithms or software that are central to the research but not yet described in published literature, software must be made available to editors and reviewers. We strongly encourage code deposition in a community repository (e.g. GitHub). See the Nature Portfolio guidelines for submitting code & software for further information.

Data

Policy information about availability of data

All manuscripts must include a data availability statement. This statement should provide the following information, where applicable:

- Accession codes, unique identifiers, or web links for publicly available datasets
- A description of any restrictions on data availability
- For clinical datasets or third party data, please ensure that the statement adheres to our policy

Source data are provided with this paper.

Research involving human participants, their data, or biological material

Policy information about studies with human participants or human data. See also policy information about sex, gender (identity/presentation), and sexual orientation and race, ethnicity and racism.

Reporting on sex and gender	This information has not been collected.
Reporting on race, ethnicity, or other socially relevant groupings	This information has not been collected.
Population characteristics	Not applicable
Recruitment	Not applicable
Ethics oversight	Not applicable

Note that full information on the approval of the study protocol must also be provided in the manuscript.

Field-specific reporting

Please select the one below that is the best fit for your research. If you are not sure, read the appropriate sections before making your selection.

☒ Life sciences ☐ Behavioural & social sciences ☐ Ecological, evolutionary & environmental sciences

For a reference copy of the document with all sections, see nature.com/documents/nr-reporting-summary-flat.pdf

Life sciences study design

All studies must disclose on these points even when the disclosure is negative.

Sample size	As described in Supplementary Information, for all experiments, results from approximately 100 or more individual samples were reported as means and standard errors. Depending on the method of analysis, such as relative values, error propagation was calculated as described in Figure S1 (Supplementary Information).<br>Previous studies (Takabe et al., 2013, Microbiology and Immunology, 10.1111/1348-0421.12018, Takabe et al., 2017 Microbiology, 10.1099/mic.0.000420, and so on) have shown that the characteristics of bacterial migration speed appear by measuring about 20 bacteria. Therefore, a lower limit of 20 bacteria was considered; however, to ensure the accuracy of the data, most experiments measured more than 100 bacteria.
Data exclusions	No data was excluded.
Replication	The number of measurements in each motility assay is the sum of more than two independent repeated experiments. The repeated experiments confirmed the reproducibility of the results. The reproducibility of immunoblot analysis shown in Figure S3 was confirmed by three independent experiments.
Randomization	The recorded bacteria were analyzed randomly and automatically by the analysis program. Bacterial clones obtained by random transposon mutagenesis were randomly picked up.
Blinding	The combination of bacterial and animal cell lines is the most important in this study. Blinding was not allowed to avoid causing mistakes in all of the experiment in this study (motility assays, immunoblot test, and mutant screening).

Reporting for specific materials, systems and methods

We require information from authors about some types of materials, experimental systems and methods used in many studies. Here, indicate whether each material, system or method listed is relevant to your study. If you are not sure if a list item applies to your research, read the appropriate section before selecting a response.

Materials & experimental systems

n/a	Involved in the study	
☐	☒	Antibodies
☐	☒	Eukaryotic cell lines
☒	☐	Palaeontology and archaeology
☐	☒	Animals and other organisms
☒	☐	Clinical data
☒	☐	Dual use research of concern
☒	☐	Plants

Methods

n/a	Involved in the study	
☒	☐	ChIP-seq
☒	☐	Flow cytometry
☒	☐	MRI-based neuroimaging

Antibodies

Antibodies used	Antibodies against leptospiral outermembrane proteins LenA and LigA were used in this study. These antibodies were raised in house. The secondary antibody used in this study is goat anti-mouse IgG (H+L) HRP conjugate (Biorad #1706516).
Validation	Data on the specificity of these antibodies were shown in this manuscript. The anibodies react specifically with their target proteins.

Eukaryotic cell lines

Policy information about cell lines and Sex and Gender in Research

Cell line source(s)	MDCK-NBL2(dog kidney cells; JCRB9029, Lot#08052019) and NRK-52E (rat kidney cells; IFO50480, Lot#09142015) were purchased from JCRB cell bank (Osaka, Japan).
Authentication	Cells were authenticated by the data sheets provided by JCRB cell bank. The cells purchased from the cell bank were first grown and stocked, and cells from the initial stock were used for each series of experiments. In addition, MDCK and NRK were discriminated by their apparently different morphology.
Mycoplasma contamination	The cell lines were not tested for mycoplasma contamination.
Commonly misidentified lines (See ICLAC register)	No commonly misidentified cell lines were used in the study

Animals and other research organisms

Policy information about studies involving animals; ARRIVE guidelines recommended for reporting animal research, and Sex and Gender in Research

Laboratory animals	A seven-week-old female BALB/c mouse was used for the production of anti-LenA antiserum.
Wild animals	*Provide details on animals observed in or captured in the field; report species and age where possible. Describe how animals were caught and transported and what happened to captive animals after the study (if killed, explain why and describe method; if released, say where and when) OR state that the study did not involve wild animals.*
Reporting on sex	*Indicate if findings apply to only one sex; describe whether sex was considered in study design, methods used for assigning sex. Provide data disaggregated for sex where this information has been collected in the source data as appropriate; provide overall numbers in this Reporting Summary. Please state if this information has not been collected. Report sex-based analyses where performed, justify reasons for lack of sex-based analysis.*
Field-collected samples	*For laboratory work with field-collected samples, describe all relevant parameters such as housing, maintenance, temperature, photoperiod and end-of-experiment protocol OR state that the study did not involve samples collected from the field.*
Ethics oversight	The production of antiserum for LenA was approved by the animal research committee of National Institute of Infectious Diseases.

Note that full information on the approval of the study protocol must also be provided in the manuscript.
